# Associations Between Attentional Bias and Interpretation Bias and Change in School Concerns and Anxiety Symptoms During the Transition from Primary to Secondary School

**DOI:** 10.1007/s10802-019-00528-3

**Published:** 2019-03-20

**Authors:** Kathryn J. Lester, Stephen C. Lisk, Ewan Carr, Fiona Patrick, Thalia C. Eley

**Affiliations:** 1 School of Psychology, University of Sussex, Pevensey Building, Brighton, BN1 9QH UK; 2 Institute of Psychiatry, Psychology and Neuroscience, King’s College London, De Crespigny Park, London, SE5 8AF UK

**Keywords:** School transition, Anxiety, Attentional Bias, Interpretation Bias

## Abstract

The transition from primary to secondary school is often associated with a period of heightened anxiety and worry. For most children, any feelings of anxiety subside relatively quickly but for a small minority, emotional difficulties can continue into the first year of secondary school and beyond. This study recruited 109 children and measured their anxiety symptoms and school concerns toward the end of primary school and again at the end of their first term of secondary school. We investigated for the first time whether pre-transition measures of attentional and interpretation bias, and the magnitude of change in attentional bias toward and away from threat stimuli were associated with pre- and post-transition measures of anxiety and school concerns, and the change in these measures over time. Over 50% of the current sample exceeded clinical levels of anxiety at pre-transition. However, anxiety symptoms and school concerns had significantly reduced by post-transition. Higher levels of pre-transition anxiety or school concerns, and a greater magnitude of change in attentional bias towards threat stimuli predicted a larger reduction in anxiety symptoms and school concerns across the transition period. A greater interpretation bias toward threat was associated with higher pre-transition anxiety symptoms and school concerns but not post-transition scores, or the change in these scores. While many children experience heightened anxiety prior to school transition, this appears to be largely temporary and self-resolves. Nonetheless, the current findings highlight the importance of monitoring children’s anxiety and concerns, and related cognitive processes during this important transition period.

**Electronic supplementary material:**

The online version of this article (10.1007/s10802-019-00528-3) contains supplementary material, which is available to authorized users.

In the UK, most children move from primary to secondary school at 11 years of age. At the time of transition, many children experience worry and anxiety, which typically subsides relatively quickly over the first term of secondary school (Rice et al. 2011; Stradling and MacNeil 2001; Zeedyk et al. 2003). For a minority of children, emotional difficulties endure, continuing into and beyond the first year of secondary education (Zeedyk et al. 2003).

School transition has been shown in some studies to negatively impact children’s emotional wellbeing (Anderson et al. 2000; Smith et al. 2008) although such studies are relatively sparse and results somewhat inconsistent (Evans et al. 2018; Grills-Taquechel et al. 2010). Nonetheless, school concerns and anxiety symptoms appear strongly correlated at primary and secondary school (Rice et al. 2011) and emotional difficulties around the transition period are associated with concurrent and prospective school attainment (Riglin et al. 2014), suggesting school transition may have a longer term negative impact on child outcomes for some. Children with enduring emotional symptoms following the school transition may represent a particularly vulnerable group (Riglin et al. 2013). The school transition period thus represents a critical time for identifying children at increased risk for poor adjustment, and for nurturing children’s mental health, especially since this developmental stage is associated with the onset of many anxiety disorders (Kessler et al. 2005).

Numerous risk and protective factors for academic, behavioural and emotional adjustment to school transition have been identified (Evans et al. 2018). Being younger, female, having lower socioeconomic status, being less academically able, and experiencing stressful life events predict greater risk for poor adjustment (Anderson et al. 2000; Rice et al. 2011; West et al. 2010). Likewise peer factors (e.g. lack of peer support, experiences of victimisation, stability in friendships) and family relationships (maternal depressive symptoms, parenting styles characterised by warmth) are also significant predictors of children’s adjustment and academic attainment (Hirsch and DuBois 1992; Ng-Knight et al. 2016; Rice et al. 2015; West et al. 2010). With regard to emotional predictors, one study showed that heightened symptoms of generalised anxiety prior to transition was associated with greater concerns about school transition both before and after transition (Rice et al. 2011). A meta-analysis also found that emotional difficulties significantly predicted poorer school attainment (Riglin et al. 2014).

There is reasonable evidence that heightened symptoms of anxiety are associated with cognitive biases in attention and interpretation that favour the selective processing of threat (Bar-Haim et al. 2007; Dudeney et al. 2015; Lau and Waters 2017; Suarez and Bell-Dolan 2001). These biases are thought to contribute causally to the onset and/or maintenance of symptoms (Van Bockstaele et al. 2014), and to impact emotional response to stressors (Hakamata et al. 2010; Osinsky et al. 2012), although this evidence is somewhat more mixed in child samples (Dodd et al. 2012; Dudeney et al. 2015). This is the first study to investigate whether attentional and interpretation biases assessed toward the end of primary school are associated with anxiety symptoms and school concerns over the school transition period.

The degree to which people respond to stressors with increases in anxiety differs substantially (Clarke et al. 2008). The basis for this variability is poorly understood but it has been argued that exposure to stressful life events may make it temporarily adaptive to develop an attentional bias for threat cues that may signal genuine danger (MacLeod 1999). As a consequence, individuals who demonstrate the greatest change in this capacity to selectively direct attention toward threat might be expected to display the most intense and sustained anxiety reactions to a subsequent stressor. Attentional bias modification studies which use experimental contingencies to encourage selective attention to threat stimuli have shown that those individuals who acquire a larger attentional bias for threat also report greater anxiety in response to a subsequent experimental stressor (MacLeod and Clarke 2015). However, findings have been more equivocal in child samples (Cristea et al. 2015).

Consistent with research using lab-based stressors, Clarke et al. (2008) found that a greater change in attentional bias toward threat stimuli (evoked using an attentional bias modification task) early in the semester predicted a larger increase in anxiety in students by the end of their first semester at university. A second study revealed that change in attentional bias away from threat was not predictive of subsequent changes in anxiety by the end of the semester. This suggests that increases in anxiety in response to mild extended stress were not explained by a high level of general attentional plasticity or a tendency to develop attentional avoidance of threat but were specifically determined by the degree to which individuals were prepared to acquire an attentional bias toward threat.

While Clarke and colleagues’ conclusions are compelling, a stronger test of their competing hypotheses requires a within-participant design where all participants are exposed to both bias contingencies (toward threat, away from threat). If individuals who show the largest change in attentional bias toward threat also show the largest change in attentional bias away from threat then this pattern might indicate that some individuals’ attentional processing is generally more malleable and responsive to environmental contingencies favouring selective attention both toward and away from threat. The inclusion of both contingency conditions also allows us to unpick whether change in anxiety symptoms/school concerns is best explained by a.) the degree to which individuals are prepared to acquire an attentional bias bias specifically towards threat, b.) the degree to which individuals acquire an attentional bias characterised by avoidance of threat, or c.) is indexed equally well by both, indicating a more general attentional malleability effect. A further rationale for including the avoid threat condition was to explore direction of effects. Some studies have shown that attentional avoidance of threat is associated with heightened anxiety (Brown et al. 2013; Stirling et al. 2006); whereas, others have shown that attentional avoidance of threat is associated with a reduction in symptoms of anxiety (e.g. Legerstee et al. 2009).

This study investigated the impact of the school transition period on children’s concerns about school and their anxiety symptoms. To do so, we recruited children during the final year of primary school and assessed the degree to which school concerns and anxiety symptoms changed between the first measurement in primary school and the second measurement at the end of the first term at secondary school. As noted previously, a small number of studies have shown that school transition negatively impacts on emotional wellbeing, although any emotional difficulties typically subside relatively quickly. In light of this, and in line with prior research (Rice et al. 2011) we began by testing the hypothesis that school concerns, and anxiety symptoms would decrease significantly over the transition period and that anxiety and school concern scores at pre-transition would correlate significantly with scores at post-transition (Hypothesis 1). Given the relatively extended nature of the school transition period and the time period between assessments in the present study, we focused on anxiety *symptoms* using the Screen for Child Anxiety Related Disorders (SCARED), rather than on trait or state anxiety per se. Prior research indicates that the SCARED taps into elements of both trait and state anxiety, but may be more strongly correlated with measures of trait anxiety (Monga et al. 2000).

Given the reasonable evidence that cognitive biases favouring the selective processing of threat are associated with heightened levels of anxiety (e.g. Lau and Waters 2017), we hypothesised that a stronger tendency to interpret ambiguous situations as threatening (Hypothesis 2) and a greater attentional bias toward threat (Hypothesis 3) prior to transition would be associated with higher school concerns, and anxiety symptoms before and after school transition. Past research has also suggested that individuals who show a greater change in their capacity to selectively direct attention toward threat (i.e. to acquire an attentional bias toward threat) but not away from threat (i.e. to acquire an attentional bias away from threat) may display more intense anxiety reactions to stressors like school transition (Clarke et al. 2008). Uniquely, we explored in the same sample whether the magnitude of change in a.) attentional bias toward threat and b.) attentional bias away from threat was associated with school concerns, and anxiety symptoms before and after school transition (Hypothesis 4). Finally, we explored whether our pre-transition measures of anxiety symptoms, school concerns, interpretation bias, attentional bias and change in attentional bias toward and away from threat predicted change in a.) school concerns and b.) anxiety symptoms across the transition period (Hypothesis 5).

## Method

### Participants

One hundred and nine children (mean age = 10.7 years, SD = 0.5, 53% female) were recruited from seven mainstream schools. Schools were invited pragmatically on the basis of being mainstream primary schools situated within a relatively broad area of Greater London but within a reasonable travelling distance using public transport from King’s College London. Ethical approval was granted by King’s College London Psychiatry, Nursing and Midwifery Research Ethics Committee (PNM/14/15–66). Parents gave written informed consent and children verbal assent. Inclusion criteria included being in the final year of primary school. Parents were advised that the study was not suitable for children with significant learning disabilities or insufficient understanding of the English language to comprehend the study materials. Forty-six percent of children identified as White British/other White background, 14% as African, and 7% as other mixed/multiple ethnic background. Fifty-five percent of parents were educated to university degree level or above. Overall the sample was more highly educated compared to the UK national average but was ethnically diverse and broadly representative with regard to ethnicity for London.

Children attended two 45-min sessions, 1 week apart during the last term of primary school. One hundred and six participants completed both sessions, and 79 participants (75% response rate) returned a follow-up questionnaire towards the end of their first term of secondary school. Compared to responders, non-responders to the follow-up questionnaire did not differ significantly on age, sex, ethnicity, anxiety symptoms, school concerns, interpretation bias or attentional bias at pre-transition (all *p* values > 0.05).

A priori power calculations were performed for the association between change in attentional bias toward threat and change in anxiety scores across the school transition. Effect size estimates were taken from Clarke et al. (2008) who observed a correlation of *r =* 0.47 between attentional bias change and anxiety change in their sample of students attending the first semester at University. With an effect size of *r* = 0.47, and to achieve 80% power with α = 0.008 (to allow for multiple testing corrections, see “Statistical Analysis” section below) required a minimum *N* = 50.

### Measures

#### Anxiety Symptoms

Anxiety symptoms (e.g. “I worry about other people liking me, When I get frightened I feel dizzy”) in the preceding 3 months were measured using the 41-item Screen for Child Anxiety Related Emotional Disorders (SCARED, (Birmaher et al. 1999). Responses were made using a 3-point scale (‘not true or hardly ever true’ – ‘very true or often true’). Internal consistency was excellent (α = 0.85 and α = 0.90 at pre- and post-transition, respectively).

#### School Concerns

Concerns about secondary school were measured using the 17-item School Concerns Questionnaire (Thomasson et al. 2006). Children rated their degree of concern for each item (e.g. ‘being bullied’, ‘following a timetable’) using a 10-point scale (1 = ‘not worried’ to 10 = ‘extremely worried’). Internal consistency was good (α = 0.92 and 0.79 at pre- and post-transition).

#### Ambiguous Situations Questionnaire – School Transition

Ten ambiguous scenarios were presented describing typical secondary school situations (see Supplementary Materials for measure development). For example, “You are in a PE lesson at your new school. Your teacher chooses two team captains and asks them to pick teams for a basketball game. You wait for your name to be called out”. Participants responded to an open-ended question designed to elicit their interpretation of the scenario (“When do you think your name will be called?”). Scenarios were presented randomly on a laptop screen using EPrime 2.0. Free choice responses were recorded, transcribed and coded using an established coding approach (K. J. Lester et al. 2010).

Participants were also presented simultaneously with a threat (“You think your name will be called near the end as the team captains won’t want you on their team”) and non-threat (“You think your name will be called very soon as the team captains will want you on their team”) forced-choice interpretation. Participants indicated which interpretation they thought was most likely to be the outcome of each situation using counterbalanced response keys. Free and forced-choice response formats were highly correlated, *r* (109) = 0.67, *p* < 0.001. Therefore, a combined threat interpretation score (range 0–100%) was computed: (total free + forced-choice threat interpretations/total number of valid interpretations) × 100.

#### Attentional Bias Change Task

This task comprised of three phases, each using a dot-probe or modified dot-probe task. The first and third phases (the pre- and post-contingency bias assessment phases) assessed relative attentional allocation to threat and neutral stimuli. The second phase (the attentional contingency block) exposed participants to a contingency designed to elicit an attentional bias favouring threat stimuli (attentional contingency – toward threat condition) or favouring neutral stimuli (attentional contingency – avoid threat condition).

Stimuli comprised of forty models (20 males and 20 females) portraying angry and neutral facial expressions selected from established face sets (Biehl et al. 1997; Langner et al. 2010; Tottenham et al. 2009). The stimuli were divided into four subsets balanced for male and female faces. The allocation of face sets and order of contingency conditions was counterbalanced using random block allocation, and blinded to the experimenter. Participants received the same face set in the pre- and post-contingency bias assessment phases (within session) and a different subset for each attentional contingency block.

**Fig. 1:**
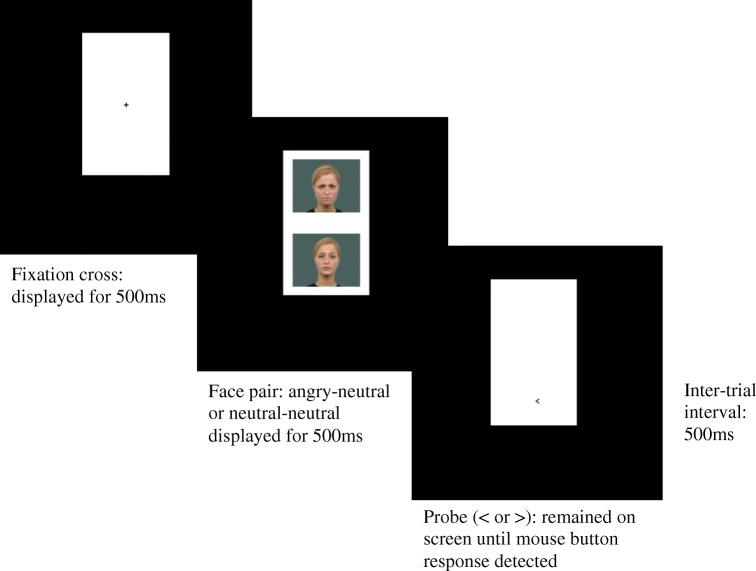
Attentional bias change task procedure

During the attentional contingency phase, participants viewed 280 trials (240 angry-neutral, 40 neutral-neutral trials). The task procedure is depicted in Fig. 1 (full details in the Supplementary Information). In the toward threat condition, the probe (< or >) consistently appeared in the location previously occupied by the angry face of angry-neutral face pairs. In the away from threat condition, the probe consistently appeared in the location previously occupied by the neutral face. The pre- and post-contingency assessment phases comprised of 120 trials (40 neutral-neutral and 80 angry-neutral trials) where the probe appeared with equal probability behind the angry and neutral stimulus. Reaction times (RTs) and accuracy of responses was recorded.

### Procedure

**Fig. 2:**
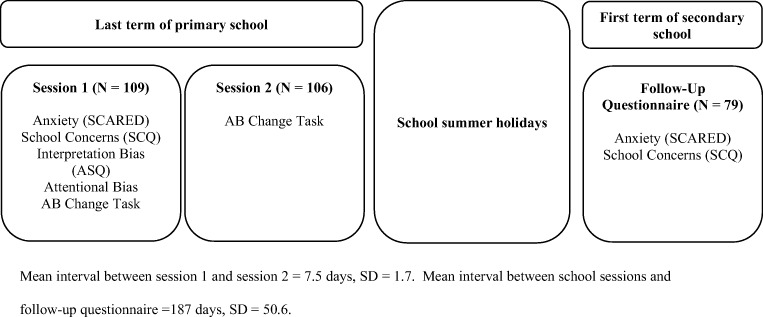
Experimental procedure

The procedure is outlined in Fig. 2. Sessions 1 and 2 were conducted in a quiet study space. In session 1, questionnaire administration was randomised, followed by completion of the ambiguous situations questionnaire, then attentional bias change task. In session 2, participants completed the opposite attentional contingency phase condition to that given in session 1. Participants received a follow-up questionnaire toward the end of their first term of secondary school. Participants received a small craft gift and a £10 gift card at the end of sessions 1 and 2, respectively, and a £10 gift card if they returned the follow-up questionnaire.

### Data Processing

Changes in school concerns (SCQ_CHANGE_) and anxiety symptoms (SCARED_CHANGE_) were computed by subtracting pre-transition scores from post-transition scores. We used an established approach to data cleaning of dot-probe reaction time data with children, and to dealing with participants with outlying responses compared to the sample mean or high error rates (Brown et al. 2014). This approach was decided upon prior to data collection and was used to remove trials with extreme (short or long) response times compared to the individuals mean reaction time, thus representing trials on which anticipatory responses were made or delayed responses, which may reflect distraction/lack of attention on that trial. Mean reaction times (RTs) were calculated for pre- and post-contingency assessments of attentional bias, after removing errors (2.8% of trials), and data values >2.5 SDs from individual means, or < 100 ms (4.1% of trials). Bias scores were calculated for pre- and post-contingency assessment phases by subtracting mean RTs for probes presented in the angry face locus from mean RTs for probes presented in the neutral face locus, resulting in a measure of participant’s attentional bias prior to contingency exposure (AB_PRE-CONTINGENCY_), and after contingency exposure (AB_POST-CONTINGENCY_). A positive score indicates a bias toward threat while a negative score indicates a bias away from threat. Participants who made incorrect or outlying responses on more than 25% of trials were excluded from analyses using bias score data. Three participants were excluded from analyses in which pre-attentional bias scores were correlated with pre- or post-transition anxiety or school concern scores or the change in those scores. Outliers comprised of participants who had mean bias scores exceeding 2.5 SD above or below the sample mean or participants who made incorrect or outlying responses on more than 25% of trials used to calculate bias scores.

Change in attentional bias (AB_CHANGE_) was computed for the “attend toward threat” condition (AB_CHANGE–TOWARD THREAT_) by subtracting AB_PRE-CONTINGENCY_ scores from AB_POST-CONTINGENCY_ scores. For the “attend away from threat” condition (AB_CHANGE–AVOID THREAT_) AB_POST-CONTINGENCY_ scores were subtracted from AB_PRE-CONTINGENCY_ scores. In both conditions larger positive scores on the AB_CHANGE_ index reflected a greater degree of change in attentional bias in the direction encouraged during the contingency phase. For analyses correlating AB_CHANGE_ scores with pre-transition measures of anxiety symptoms or school concerns, eight participants were excluded due to having mean change scores exceeding 2.5 SD above or below the sample mean or because they made incorrect or outlying responses on more than 25% of trials used to calculate bias scores. For analyses correlating AB_CHANGE_ scores with post-transition measures of anxiety symptoms or school concerns or change in these outcomes, six participants were excluded for the same reasons. After data processing all variables approximated a normal distribution. Sensitivity analyses were conducted in which participants who were outliers for attentional bias scores or for AB_CHANGE-TOWARD THREAT_ or AB_CHANGE-AVOID THREAT_ scores were not removed from analyses. Overall, the substantive conclusions remained unchanged despite minor fluctuations in effect size and *p* value (see Supplementary Materials).

### Statistical Analysis

We examined bivariate correlations between all variables both within and across time points and between anxiety symptom and school concern change scores and all pre-transition variables. Bonferroni corrections were applied to control for the number of tests performed. Changes in anxiety symptoms and school concerns were analysed using ordinary least squares (OLS) linear regression. Assumptions for OLS regression were carefully checked. First, scatterplots were checked and showed that relationships between the independent and dependent variables were linear. Second, there was no evidence of multicollinearity (correlations between independent variables were all lower than 0.8 and variance inflation factors ranged 1.0–1.9). Third, plots of standardised residuals vs. standardised predicted values showed no obvious signs of funnelling suggesting the assumption of homoscedasticity was met. Fourth, all Cook’s distance values were under 1 (range 0.0–0.5) suggesting that no individual cases were unduly influencing the model. Finally, normal probability plots of the standardised residuals for each model indicated some mild deviations from normality. While mild deviations from normality are unlikely to impact on the validity of our findings, as a sensitivity analysis we re-ran the regression models using bootstrapped 95% bias corrected and accelerated confidence intervals (with 1000 bootstrap iterations). These analyses gave results that were consistent to the models without bootstrapping and did not change our substantive conclusions (full results available on request).

We included as covariates pre-transition variables that were significantly associated with the outcome (SCARED_CHANGE_ and SCQ_CHANGE_) in the bivariate correlations (*p* < 0.05). When a significant covariate is identified, it is significant over and above the other covariates in the model. In all analyses involving SCARED_CHANGE_ and SCQ_CHANGE_ scores we controlled for the pre-transition level of the outcome. For the change score Δ*Y* = *Y*_2_ − *Y*_1_, we include Y_1_ as a covariate in the model; for bivariate correlations, we estimate partial correlations, controlling for Y_1._ Controlling for pre-transition scores is important because of correlations between pre-transition scores on the outcome and exposure (i.e. Y_1_ and X_1_). Pre-transition outcome scores (Y_1_ e.g. pre-transition SCARED scores) are therefore correlated with both pre-transition exposures (X_1_ e.g. interpretation bias scores) and the post-transition outcome (Y_2_ e.g. post-transition SCARED scores). This represents a form of confounding that must be controlled for (Pearl 2016). Failure to account for correlations between Y_1_ and X_1_ will lead to biased results. This issue only arises when Y_1_ and X_1_ are correlated (i.e. when Y_1_ predicts both X_1_ and ΔY (e.g. SCARED_CHANGE_ score), and thus, confounds the association between X_1_ and ΔY). Where X_1_ and Y_1_ are uncorrelated (or are weakly correlated), there is no confounding due to Y_1_, and the results are not affected by controlling for Y_1_. However, for consistency, all estimates have been adjusted for baseline outcome scores (Y_1_).

## Results

### Hypothesis 1: Correlations Between Anxiety Symptoms and School Concerns at Pre- and Post-Transition

We hypothesised that school concerns and anxiety symptoms would decrease significantly over the transition period and that anxiety and school concern scores at pre-transition would correlate significantly with scores at post-transition. Pre-transition mean scores for anxiety and school concerns (see Table 1) were approximately 0.5 SD above reported norms while scores at post-transition were comparable to previous reports for school concerns (Rice et al. 2011) and were 0.25 SD below the norms for non-anxiety cases (Birmaher et al. 1999). Consistent with this hypothesis, we observed a significant reduction in school concerns and anxiety symptoms from pre- to post-transition (Table 1). Nonetheless, there was substantial inter-individual variability. Anxiety symptoms and school concerns were moderately correlated at pre- and post-transition (see Table 2) and, consistent with our hypothesis, there was continuity such that individuals with higher scores at pre-transition also reported higher scores at post-transition.

**Table 1:** Descriptive and test statistics for school concerns, and anxiety symptoms at pre- and post-school transition

Measure	Pre-transition	Post-transition	Change score	*t* (df)	*p*
	Mean (SD)	Mean (SD)	Mean (SD) [95% CI]		
School concerns (SCQ)	78.21 (32.36) (*n* = 106)	46.30 (24.58) (*n* = 78)	−29.32 (27.97) [−35.58 – −23.05]	−9.32 (78)	< 0.001
Anxiety symptoms (SCARED)	26.70 (14.72) (*n* = 106)	16.10 (13.79) (*n* = 76)	−9.17 (12.51) [−12.01 – −6.33]	−6.43 (76)	< 0.001
Interpretation Bias (ASQ_PRE_)	32.93 (19.13) (*n* = 109)	–	–	–	–
Attentional Bias (S1 AB_PRE-CONTINGENCY_)	−3.63 (36.19) (*n* = 106)	–	–	–	–
AB_CHANGE – TOWARD THREAT_	−0.05 (51.45) (*n* = 101)	–	–	–	–
AB_CHANGE – AVOID THREAT_	−6.04 (56.43) (*n* = 98)	–	–	–	–

*SCQ* School Concerns Questionnaire, *SCARED* Screen for Child Anxiety Related Emotional Disorders, *ASQ* Ambiguous Situations Questionnaire, *AB* Attentional Bias

**Table 2:** Bivariate correlations between school concerns, anxiety symptoms and cognitive measures at pre- and post-school transition

	SCARED_PRE_	SCQ_POST_	SCARED_POST_	SCQ_CHANGE_	SCARED_CHANGE_	ASQ_PRE_	S1 AB_PRE-CONTINGENCY_	AB_CHANGE-TOWARD THREAT_	AB_CHANGE-AVOID THREAT_
SCQ_PRE_	**0.687**	**0.509** ^**a**^	**0.024** ^**ab**^	**-0.609** ^**a**^	−0.022 ^ab^	**0.542**	0.013	−0.053	−0.053
	**<0.001**	**<0.001**	**<0.001**	**<0.001**	0.851	**<0.001**	0.895	0.600	0.606
SCARED_PRE_	–	0.250^ab^	**0.633** ^**a**^	0.229^ab^	**-0.447** ^**a**^	**0.533**	−0.140	−0.012	−0.219
		0.030	**<0.001**	0.047	**<0.001**	**0.000**	0.153	0.908	0.030
SCQ_POST_		–	**0.672**	–	–	-0.187^ab^	0.119^ab^	-0.159^ab^	-0.233^ab^
			**<0.001**			0.106	0.309	0.189	0.054
SCARED_POST_			–	–	–	-0.170^ab^	0.123^ab^	-0.314^ab^	-0.076^ab^
						0.146	0.300	0.009	0.540
SCQ_CHANGE_				–	–	-0.153^ab^	0.065^ab^	-0.277^ab^	0.009^ab^
						0.186	0.579	0.020	0.944
SCARED_CHANGE_					–	-0.079^ab^	0.147^ab^	**-0.392** ^**ab**^	0.078^ab^
						0.502	0.215	0**.001**	0.531

*SCQ* School Concerns Questionnaire, *SCARED* Screen for Child Anxiety Related Emotional Disorders, *ASQ* Ambiguous Situations Questionnaire, *AB* Attentional Bias. ^a^Partial correlation coefficients controlling for interval in days between in-school sessions and return of follow-up questionnaires.^b^Partial correlation coefficients controlling for interval in days between in-school sessions and return of follow-up questionnaires, and pre-transition level of the outcome. Correlation coefficients in bold survive multiple testing corrections: Bonferroni corrected *p* value for correlations with SCARED_PRE_ and SCQ_PRE_ = 0.01; for correlations with SCARED_POST_ and SCQ_POST_ = 0.008; for correlations with SCARED_CHANGE_ and SCQ_CHANGE_ = 0.008

### Hypothesis 2: Associations Between Interpretation Bias and Anxiety Symptoms and School Concerns at Pre- and Post-Transition

We hypothesised that a greater interpretation bias favouring threat would predict higher levels of anxiety symptoms and school concerns at pre- and post-transition. In support of this hypothesis we found that a stronger tendency toward interpreting ambiguous situations as threatening was significantly associated with greater school concerns and anxiety symptoms prior to school transition (see Table 2). However threat interpretation bias did not significantly correlate with anxiety symptoms or school concern scores at post-transition.

### Hypothesis 3: Associations Between Attentional Bias and Anxiety Symptoms and School Concerns at Pre- and Post-Transition

We hypothesised that a greater attentional bias for threat would predict higher levels of anxiety symptoms and school concerns at pre- and post-transition. The mean bias score was −3.6 (SD = 36.2) indicating no significant attentional bias in either direction (difference from 0, *t* (109) = −1.03, *p* = 0.304). There were substantial individual differences in bias scores (range: −127.33 to 120.18). Contrary to our hypothesis, however, pre-transition attentional bias was not significantly associated with pre-or post-transition anxiety symptom or school concern scores (see Table 2).

### Hypothesis 4: Associations Between Attentional Bias Change and Anxiety Symptoms and School Concerns at Pre- and Post-Transition

We tested the hypothesis that the magnitude of change in attentional bias toward threat (AB_CHANGE–TOWARD THREAT_) and magnitude of change in attentional bias away from threat (AB_CHANGE–AVOID THREAT_) would be associated with school concerns and anxiety symptoms at pre- and post-transition. AB_CHANGE– TOWARD THREAT_ scores were not significantly related to pre-transition school concerns or anxiety symptoms (see Table 2). Larger changes in attentional bias toward threat were significantly associated with lower anxiety symptoms at post-transition (but not school concerns), although this effect did not survive multiple testing corrections. AB_CHANGE–AVOID THREAT_ scores were unrelated to pre-transition school concerns. However, higher anxiety symptoms at pre-transition was significantly associated with a smaller magnitude of attentional bias change away from threat, although this too was no longer statistically significant after corrections for multiple testing. No significant associations were observed with post-transition school concerns, or anxiety symptoms. No significant correlation was observed between AB_CHANGE–TOWARD THREAT_ and AB_CHANGE–AVOID THREAT_ scores (*r* (92) = −0.17, *p* = 0.110).

### Hypothesis 5: Correlates and Prediction of Change in Anxiety Symptoms and School Concerns from Pre-Transition Variables

Our final hypothesis investigated whether pre-transition assessments (of anxiety symptoms, school concerns, interpretation bias, attentional bias and change in attentional bias toward and away from threat**)** predicted change in school concerns and anxiety symptoms over the transition period. Overall, we found good support for this hypothesis. Greater school concerns at pre-transition was significantly associated with a larger reduction in school concerns over the transition period (see Table 2) with the same pattern observed between anxiety scores. No significant correlations were observed between pre-transition measures of interpretation bias, attentional bias, or AB_CHANGE-AVOID THREAT_ scores and SCQ_CHANGE_ and SCARED_CHANGE_ scores. However, a greater change in attentional bias toward threat stimuli (AB_CHANGE-TOWARD THREAT_) was significantly associated with a larger reduction in school concerns and anxiety symptoms over the transition period. The correlation with SCQ_CHANGE_ did not survive multiple testing corrections.

To test hypothesis 5, changes in school concerns and anxiety symptoms were analysed using multiple regression. Only pre-transition variables that were significantly associated with the outcome (SCARED_CHANGE_ and SCQ_CHANGE_) in the bivariate correlations reported above (*p* < 0.05) were included in each model. Change in school concerns was significantly associated with pre-transition school concerns, pre-transition anxiety symptoms and AB_CHANGE-TOWARD THREAT_ scores (see Table 3). Higher pre-transition school concerns significantly predicted a reduction in school concerns scores across the transition period (β = −0.75). Higher pre-transition anxiety scores were positively associated with SCQ_CHANGE_ (β = 0.28). Increases in attentional bias toward threat predicted a decrease in school concerns across the transition period. A one SD increase in AB_CHANGE–TOWARD THREAT_ scores (51.2ms) predicted a reduction of 7 points on the SCQ between pre- and post-transition (β = −0.24; *p* = 0.009).

**Table 3:** Regression analyses for change in school concerns and anxiety symptoms

	SCQ_CHANGE_				SCARED_CHANGE_			
	*b* [95% CI]	β	*t*	*p*	*b* [95% CI]	β	*t*	*p*
SCQ_PRE_	−0.75 [−0.97 – −0.52]	−0.81	−6.69	<0.001	–	–	–	–
SCARED_PRE_	0.59 [0.08–1.10]	0.28	2.32	0.023	−0.39 [−0.57 – −0.22]	−0.45	−4.47	<0.001
AB_CHANGE-TOWARD THREAT_	−0.14 [−0.24 – −0.04]	−0.24	−2.69	0.009	−0.09 [−0.13 – −0.04]	−0.35	−3.49	0.001

*SCQ* School Concerns Questionnaire, *SCARED* Screen for Child Anxiety Related Emotional Disorders, *AB* Attentional Bias

Change in anxiety symptoms was significantly predicted by pre-transition anxiety symptoms and AB_CHANGE-TOWARD THREAT_ scores. Higher pre-transition anxiety predicted a greater reduction in anxiety symptoms across the transition period. A one SD increase in pre-transition anxiety score (14.23) predicted a reduction of 6 points on the SCARED between pre- and post-transition (β = −0.45; *p* < 0.001). Likewise, a one SD increase in AB_CHANGE-TOWARD THREAT_ scores (51.2ms) predicted a reduction of 4 points on the SCARED between pre- and post-transition (β = −0.35; *p* = 0.001).

## Discussion

This study explored whether attentional biases, the malleability of attentional biases, and interpretation bias measured before school transition explained individual differences in changes in anxiety symptoms and school concerns over the transition period. In support of hypothesis 1, we observed a significant decrease in anxiety symptoms and school concerns from pre- to post-transition. Anxiety and school concern scores at pre-transition also correlated significantly with post-transition scores. Hypothesis 2 was also partly supported with a greater threat interpretation bias associated with higher pre-transition anxiety symptoms and school concerns but not post-transition scores. Hypothesis 3 was not supported as no significant associations were observed between pre-transition attentional bias and pre- or post- anxiety or school concern scores. We also found no convincing support for hypothesis 4; attentional bias change toward and away from threat were not significantly correlated with pre- or post- transition anxiety symptom or school concern scores after multiple testing corrections were applied. In support of hypothesis 5, we identified a small number of significant predictors of change in anxiety symptoms and school concerns across the transition period. Higher levels of pre-transition anxiety or school concerns were associated with greater reductions in severity across transition. A larger increase in attentional bias toward threat significantly predicted a larger reduction in anxiety symptoms and school concerns across the school transition period.

The period towards the end of primary school appears to be an especially stressful time with around half of our sample exceeding the suggested clinical cut-off on the SCARED. We also observed continuity of symptoms, such that higher anxiety symptoms at pre-transition were correlated with retaining heightened anxiety symptoms at post-transition. This is consistent with prior research showing that anxiety symptoms in primary school predict later symptoms (Lester et al. 2013). Higher anxiety scores pre-transition predicted a decrease in anxiety scores over time. The same effects were observed for school concerns. This pattern of results may reflect a combination of regression toward the mean and participants with higher pre-transition scores having greater room to shift downwards on each measure over time. However, while children with higher anxiety at pre-transition appeared to have a steeper slope of change in anxiety over the time period, they still retained higher levels of anxiety symptoms at post-transition.

For most children heightened symptoms of anxiety and school concerns were relatively short-lived, with mean scores significantly reduced by post-transition relative to pre-transition. One possible explanation is that by the end of their first term of secondary school, most children had adjusted to changes in the physical and personal school environment, in turn leading to reductions in anxiety symptoms and school concerns. However, changes in these measures may also be accounted for by other processes that occur in the time interval between the end of primary school, and the start of secondary school that are independent of the transition to a new school (Lohaus et al. 2004). Lohaus and colleagues suggest that the reduction in stress and symptoms observed may reflect a recovery effect over the summer break from school. This recovery effect may outweigh any possible stress-inducing effects of the transition for the majority of children resulting in the observed reductions in anxiety symptoms and school concerns by post-transition. However, there was a subset of children with elevated anxiety symptoms at pre-transition who continued to experience heightened anxiety post-transition even after any recovery effect of the summer holidays. Around half of children who exceeded the clinical cut-off for anxiety at pre-transition retained clinically severe anxiety levels at post-transition. Furthermore, 22.1% and 29.9% of the current sample reported either no change or an increase in anxiety symptoms, or school concerns by post-transition.

A larger change in attentional bias toward threat significantly predicted a greater reduction in anxiety symptoms and school concerns across time. This was unexpected and at odds with prior research that observed a larger change in attentional bias toward threat at the start of the first semester of university predicted a greater increase in anxiety by the end of the semester (Clarke et al. 2008). It is argued that this is because a heightened preparedness to acquire a threat bias in response to an experimental contingency that favours selective threat processing predicts who will naturally develop an attentional bias for threat when exposed to a real-life extended stressor, in turn predicting who will experience increases in anxiety. However, unlike the young adults who transitioned to university, children who transitioned to secondary school reported a significant reduction in anxiety over time. Notably, the current sample of children were considerably more anxious at the study outset compared to the sample of young adults in Clarke et al. (2008). A later study assessed change in attentional bias toward threat among individuals with social anxiety disorder prior to cognitive behaviour therapy (Clarke et al. 2012). They also found, as we did with children, that in a sample of adults with initially elevated anxiety levels, participants with the largest change in attentional bias toward threat showed the greatest reductions in anxiety across treatment. They argue that this is because preparedness to acquire a threat bias reflects a general attentional plasticity effect whereby individuals who most readily acquire a bias favouring threat in response to a contingency making a threat bias adaptive will also be most likely to adopt the reverse processing bias when exposed to environmental conditions such as therapy, which make this adaptive and so reduce anxiety. However, our findings that readiness to acquire a bias toward and away from threat were not correlated and change in anxiety not predicted by change in bias away from threat argues against such a general plasticity account. Further research is needed to better understand the differences in patterns of effects seen, and to fully explain the mechanisms underpinning why preparedness to acquire a threat attentional bias was adaptive in this instance (and in Clarke et al. 2012) but predicted maladaptive increases in anxiety in Clarke et al. (2008).

CBT for social anxiety and school transition have in common the fact that on average participants begin with elevated anxiety levels which decrease over the course of treatment/time. Furthermore, the process of CBT for social anxiety and arguably the school transition experience typically involve some form of repeated exposure involving confrontation with a feared object, situation, or anxiety-provoking thought. The content of fears around changes in social environment and relationships/experiences with others may also be similar for individuals receiving CBT for social anxiety, and children transitioning to secondary school. Within CBT, and perhaps during school transition, successive exposures to a feared stimuli or situation in the absence of any aversive consequences, should result in the individual learning that their feared object is not predictive of an aversive outcome and ultimately anxiety is reduced (Craske et al. 2014). An enhanced capacity to attend to, identify and engage with threat as a consequence of more readily acquiring an attentional bias for threat might increase the opportunity for an individual to learn that their feared object is not necessarily predictive of an aversive outcome, thus facilitating extinction of fears and reducing anxiety (Barry et al. 2015). A small number of studies have indeed shown that a stronger tendency to attend to threat relative to attending away from threat or no bias is predictive of improved response to exposure treatment (Legerstee et al. 2010; Price et al. 2011; Waters et al. 2012). Similar processes may account for the present pattern of findings: A greater readiness to acquire an attentional bias toward threat may have facilitated children learning that a potentially feared and threatening school situation was not necessarily predictive of something aversive occurring leading to a reduction in anxiety symptoms and school concerns.

Change in attentional bias away from threat was not significantly associated with change in anxiety or school concerns. The correlation between change in anxiety symptoms and change in attentional bias away from threat was in the opposite direction and was significantly different from the correlation coefficient between change in anxiety and attentional bias change toward threat (*Z* (57) = −2.18, *p* = 0.032). This suggests that reductions in anxiety across transition were specifically predicted by the degree to which individuals acquired an attentional bias toward threat and not by a high level of general attentional plasticity. Measures of change in attentional bias toward and away from threat were not significantly correlated. This is inconsistent with a small number of studies which have observed that malleability in attentional bias toward and away from threat are equally predicted by other factors, including variation in the 5HTTLPR gene (Fox et al. 2011) and a measure of attentional control (Basanovic et al. 2017).

When faced with ambiguous school situations, children with heightened anxiety and school concerns at pre-transition were more likely to resolve that ambiguity in a threatening way. Interpretation bias was not significantly associated with anxiety or school concerns at post-transition or the change in these measures. Our findings are more consistent with interpretation bias being a consequence or epiphenomenon of anxiety (Dodd et al. 2012), and with prospective studies, which have found no or minimal significant evidence for a longitudinal relationship between interpretation bias and anxiety symptoms (Creswell et al. 2011; Dodd et al. 2012; Muris et al. 2004).

Pre-transition attentional bias for threat was not significantly associated with anxiety symptoms and school concerns, or the change in these measures. There is very limited research investigating prospective associations between attentional biases and anxiety in child samples (Morales et al. 2015), and there is evidence to suggest that attentional biases in children may only be observed at clinical levels of anxiety (Bar-Haim et al. 2007; Dudeney et al. 2015). We found only very minimal evidence for any association with anxiety symptoms even when we confined our analyses to those children exceeding the clinical cut-off. A recent meta-analysis found the effect size for the association between child anxiety and attentional bias was smaller compared to adults, less robust, and sensitive to important methodological details such as task type and format (Dudeney et al. 2015).

This study has several limitations. We had no control group of non-transitioning children and only two assessment points. With three or more measurements we could have tracked changes in symptoms more precisely, and could also have explicitly modeled the correlation between intercept and slope of change rather than simply controlling for pre-transition outcome scores. With a relatively modest N of 79, we were 80% powered to detect a correlation of 0.31. While most of the key findings in our study exceeded this effect size, we were underpowered to detect smaller effects, and Type II errors may be present. We did not have diagnostic measures or data on treatment use, and cannot discount the possibility that diagnostic and treatment status may have moderated the association between attentional and interpretation bias and symptom change across time. We used a measure of anxiety symptoms assessed over a 3-month period rather than separate measures of state and trait anxiety. The SCARED likely taps elements of both trait and state anxiety but appears to more highly correlated with trait anxiety (Monga et al. 2000). However, we were unable to unpick the impact of school transition on state and trait anxiety independently or to fully investigate whether participants with high trait anxiety differ in their trajectory and correlates of change from those participants who reported only being high state but not high trait anxious. Notwithstanding this, the findings in the subset of the sample exceeding clinical cut-off scores for anxiety were very similar in direction and magnitude of effects to those observed in the entire sample (see Supplementary Materials). There are many other factors that we could have measured using not only child but also parent or teacher-report measures, (e.g. stressful life events, peer relationships, bullying and victimisation, parenting styles and psychopathology) and which may predict emotional adjustment across school transition directly, or indirectly by influencing change in children’s attentional responses to emotional stimuli.

For most children there were no persistent negative effects of school transition. However, a concerning proportion of children reported clinical levels of anxiety, and importantly, higher anxiety symptoms at pre-transition were associated with the retention of higher anxiety following transition. These findings reiterate the importance of monitoring children’s emotional wellbeing during a time of heightened stress, which corresponds with a sensitive period for the development of anxiety disorders. This is the first study to demonstrate an association between magnitude of change in attentional bias toward threat and change in anxiety symptoms and school concerns in response to school transition. More research is needed to unravel the mechanisms underpinning this association, and to determine whether this relationship can be exploited in intervention approaches.

## Electronic supplementary material

ESM 1 (DOCX 45.8 kb)
