# Supplementary material for: Associations Between Attentional Bias and Interpretation Bias and Change in School Concerns and Anxiety Symptoms During the Transition from Primary to Secondary School
Source: J Abnorm Child Psychol. 2019 Mar 20;47(9):1521–32. doi: 10.1007/s10802-019-00528-3 (PMC6647860; doi:10.1007/s10802-019-00528-3)
Supplement: Supplementary file 1 — (DOCX 45.8 kb) [file 10802_2019_528_MOESM1_ESM.docx]

# Supplementary Materials

## Development of the Ambiguous Situations Questionnaire – School Transition

Eighteen ambiguous situations related to situations that children may experience following the transition to secondary school were developed using themes from the School Concerns Questionnaire. Twelve independent raters provided feedback and pilot ratings on each item. This comprised of generating possible interpretations for each scenario, commenting on wording, and rating the forced choice responses for threat content and plausibility. Raters were presented with each situation, and the corresponding question and forced choice threat and non-threat interpretations for each situation. They rated how threatening each interpretation was on a four-point scale (not at all threatening – very threatening) and how plausible it was as an interpretation/response to the situation described (not at all plausible – very plausible). For each situation, paired t-tests were performed to compare the threat and non-threat interpretations on ratings of threat and plausibility. Ten situations were selected for the final version of the questionnaire where the threat interpretation was rated as significantly more threatening in content than the non-threat interpretation and there was no significant difference in plausibility rating between the threat and non-threat interpretations. For these ten items, threat interpretations were rated as significantly more threatening than non-threat interpretations (2.89 vs. 1.26, *t* (11) = -7.73, *p* < .001) but both interpretations were rated as equally plausible (280 vs. 2.51, *t* (11) = -1.65, *p* = .13.

## Coding of the Ambiguous Situations Questionnaire – School Transition

Responses were independently coded by an undergraduate researcher to determine whether the response reflected a threat or non-threat interpretation, ambiguous or missing response. Where the child gave more than one possible interpretation in their response only the first interpretation was coded. A second undergraduate student coded a randomly selected subset of responses (30%) with inter-rater agreement of k = .82, p <.001.

**Description of Attentional Change Task**

During the attentional contingency phase, participants viewed 280 trials (240 angry-neutral trials, 40 neutral-neutral trials) of a modified dot probe task. Each trial began with the presentation of a fixation display (500ms; white cross 1*1 cm at the centre of the screen), on which the participants were requested to focus their gaze. The fixation display was followed by a face pair display (500ms). Participants were presented with pairs of faces of the same actor (e.g. neutral angry), which were 45mm in width and 34mm in height, and presented equidistant from the top and bottom of the fixation cross with a distance of 14mm between them. The face stimuli were displayed on a black background screen with the face stimuli surrounded by a single 58mm wide by 94mm tall white rectangle denoting the general area on the screen on which to focus. Immediately following the faces display, a target probe appeared consisting of an arrowhead pointing either left or right (< or >) in the location previously occupied by one of the faces. Participants were required to determine which probe appeared by pressing one of two specified mouse buttons with the probe remaining on screen until a response was detected. The inter-trial interval was 500ms. In the toward threat condition, the probe consistently appeared in the location previously occupied by the angry face of angry-neutral face pairs, thereby encouraging the emergence of individual differences in the adoption of a bias favouring selective attention to threat. In the away from threat condition, the probe consistently appeared in the location previously occupied by the neutral face of angry-neutral face pairs, thereby encouraging the emergence of individual differences in the adoption of a bias favouring selective attention away from threat and toward neutral stimuli. Participants received a short break every 40 trials. When accuracy in the preceding block fell below 70%, a warning slide was presented during the break, providing an opportunity for the experimenter to remind the participant not to compromise accuracy. This phase took approximately 14 minutes to complete.

The pre- and post-contingency assessment phases comprised of 120 trials of which 40 were neutral-neutral trials and 80 angry-neutral trials where the probe appeared with equal probability behind the angry and neutral stimulus. Each phase took approximately 6 minutes to complete. Reaction times (RTs) and accuracy of responses were recorded.

## Age and Sex Effects on Anxiety Symptoms and School Concerns at Pre- and Post-Transition

No significant correlations with age were observed for any measures at pre- or post- transition time points. Girls reported significantly higher pre transition school concerns than boys (*t* (107) = 3.41, *p* = .001; 87.67 (30.53) vs. 67.45 (31.27). This was also the case for anxiety symptoms (*t* (107) = 3.69, *p* < .001; 31.31 (14.97) vs. 21.45 (12.65). No significant difference between boys and girls was observed at post-transition for school concerns (*p* = .260), or anxiety symptoms (*p* = .125).

## Attentional Bias Change – Effect on Attentional Bias at a Sample Level

To determine the impact of the attentional bias contingency phase on the change in attentional bias toward and away from threat stimuli, mean RTs were entered into a mixed ANOVA with time (pre, post) and Probe valence (probe behind neutral, probe behind threat stimulus) as within subjects variables and Order (threat (neutral) contingency condition received 1^st^ or 2^nd^) as a between subjects variable. This analysis was performed separately for each attentional contingency condition. The critical Time x Probe Valence interaction was non-significant for the toward threat contingency condition (*F* (1, 98) = 0.07, *p* = .790). For the avoid threat contingency condition, there was a significant Time x Probe Valence x Order interaction (*F* (1, 94) = 4.96, *p* = .028). To interpret this effect, we independently tested the Time x Probe Valence interaction in the subset of the sample who received the avoid threat contingency condition first and second respectively. For those who received the avoid threat contingency condition second, the critical Time x Probe valence interaction was non-significant. However, the Time x Probe valence interaction was significant for those who received the avoid threat contingency condition first (*F* (1, 43) = 5.77, *p* = .021. Participants were significantly faster to respond to probes that appeared behind both neutral and threat stimuli at post evocation compared to pre evocation. However, contrary to expectations, the magnitude of this speeding in response was larger when the probe appeared behind threat (pre: 611.03ms post: 559.18ms) stimuli relative to neutral stimuli (pre: 602.79 post: 571.12). Overall, at a sample level, the attentional bias contingency phase was ineffective at evoking an attentional bias toward or away from threat stimuli.

## Variability in the Magnitude of Change in Attentional Bias to Emotional Stimuli

Variability in AB_CHANGE – TOWARD THREAT_ ranged from -153.76ms to a maximum of 141.17ms (mean = -0.05ms, SD = 51.45). AB_PRE-CONTINGENCY_ scores were significantly correlated with AB_POST-CONTINGENCY_ scores (*r* (101) = .22, *p* = .031), although only 5% of the variance was shared. This indicates attentional responses to the threat condition were highly variable. AB_PRE-CONTINGENCY_ scores were also significantly correlated with AB_CHANGE – TOWARD THREAT_ scores (*r* (101) = -.44, *p* < .001) indicating that a weaker attentional bias toward threat in the pre-contingency phase was associated with greater magnitude of change in attentional bias toward threat.

Variability in AB_CHANGE – AVOID THREAT_ ranged from -168.70ms to a maximum of 169.48ms (mean = -6.04ms, SD = 56.43). AB_PRE-CONTINGENCY_ scores were not significantly correlated with AB_POST-CONTINGENCY_ scores (*r* (98) = .097, *p* = .341), suggesting substantial individual differences in the effectiveness of the neutral contingency condition. AB_PRE-CONTINGENCY_ scores were also significantly correlated with AB_CHANGE – AVOID THREAT_ scores (*r* (98) = .574, *p* < .001) indicating that a weaker attentional bias toward neutral stimuli in the pre-contingency phase was associated with greater magnitude of change in attentional bias toward neutral stimuli.

**Sensitivity Analyses**

Sensitivity analyses were conducted in which participants who were outliers for attentional bias scores or for AB_CHANGE-TOWARD THREAT_ or AB_CHANGE-AVOID THREAT_ scores were not removed from analyses. All correlations with pre- and post-transition anxiety symptom and school concern scores, and the change in these outcomes showed the same direction of effects compared to the analyses with outliers removed. The magnitude and significance of the associations also remained very similar. All associations that were nonsignificant (taking into account multiple testing corrections) in the analyses with outliers removed were also non-significant in the sensitivity analyses, aside from one exception. The association between AB_CHANGE-TOWARD THREAT_ and post-transition SCARED score had a *p* value of .009 (*r* = -.314) in the analyses with outliers removed (and thus did not survive multiple testing correction), compared to *p* = .002 (*r* = -.348) in the sensitivity analyses. Additionally, the significant association between AB_CHANGE-TOWARD THREAT_ and SCARED_CHANGE_ score (*p*  = .001) was only nominally significant in the sensitivity analyses (*p* = .027). Sensitivity analyses were also performed for the regression analyses predicting change in anxiety symptom and school concern scores. For the anxiety symptom analyses, the direction and significance of effects were highly similar to the analyses with outliers removed. For the school concern analyses, increases in attentional bias toward threat predicted a decrease in school concerns across the transition period, but this effect was no longer statistically significant (*t =* -1.37, *p* = .18). Overall, the substantive conclusions remained unchanged despite minor fluctuations in effect size and *p* value.

**Exploratory Analyses Confined to Individuals Exceeding Clinical Cut-off for Anxiety Symptoms**

We conducted exploratory analyses confined to the subset of the sample with anxiety symptom scores exceeding the suggested clinical cut-off (Birmaher et al., 1999). Pre-transition anxiety symptoms, and school concern scores were significantly correlated with interpretation bias (*r =* .503, *p* < .001 and *r =* .436, *p* < .001). The attentional bias score observed in this subset did not differ significantly from 0 (*t* (52) = -0.615, *p* = .541) and did not differ from those scoring below the suggested cut-off (*t* (104) = .039, *p* = .969). We observed a small association between higher anxiety symptoms and attentional avoidance of threat (*r* = -.270, *p* = .05) at pre-transition only and not with any other measures. Change in anxiety symptom scores was significantly associated with change in attentional bias toward threat stimuli only, with the direction of effects the same as that observed in the full dataset (*r* = -.418, *p* = .030). Change in school concerns was not significantly associated with any variables in this subset.
